# (4-Hydroxy­phen­yl)methanaminium 2-(4-sulfanylphen­yl)acetate

**DOI:** 10.1107/S1600536809034540

**Published:** 2009-09-05

**Authors:** Ying-Jie Cai, Xi-Bin Dai, Lian Liu, Jin Li, Hai-Yan Li

**Affiliations:** a Engineering Research Center for Clean Production of Textile Dyeing and Printing, Ministry of Education, Wuhan 430073, People’s Republic of China

## Abstract

In the title mol­ecular salt, C_7_H_10_NO^+^·C_8_H_7_O_2_S^−^, the crystal structure is stabilized by inter­molecular N—H⋯O, O—H⋯N and C—H⋯O hydrogen bonds.

## Related literature

For related mol­ecular salts, see: Xia *et al.* (2003 ▶); He *et al.* (2008 ▶). For reference structural data, see: Allen *et al.* (1987 ▶).
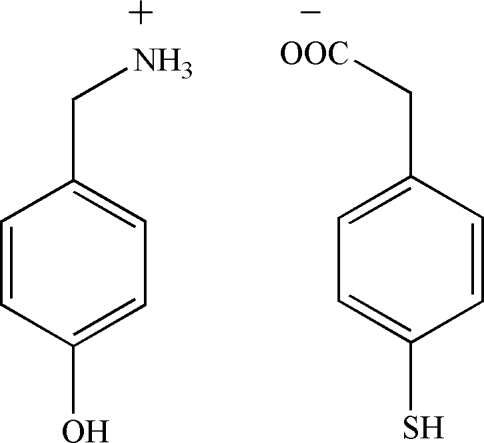


## Experimental

### 

#### Crystal data

C_7_H_10_NO^+^·C_8_H_7_O_2_S^−^
*M* *_r_* = 291.37
Monoclinic, 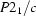

*a* = 6.545 (5) Å
*b* = 14.792 (12) Å
*c* = 14.868 (11) Å
β = 104.78 (4)°
*V* = 1391.8 (19) Å^3^
*Z* = 4
Mo *K*α radiation
μ = 0.24 mm^−1^
*T* = 293 K
0.32 × 0.28 × 0.26 mm

#### Data collection

Enraf–Nonius CAD-4 diffractometer
Absorption correction: ψ scan (North *et al.*, 1968 ▶) *T*_min_ = 0.927, *T*_max_ = 0.940
6838 measured reflections
2447 independent reflections
1895 reflections with *I* > 2σ(*I*)
*R*_int_ = 0.061
??? standard reflections every ??? reflections intensity decay: ???%

#### Refinement

*R*[*F* ^2^ > 2σ(*F* ^2^)] = 0.054
*wR*(*F* ^2^) = 0.182
*S* = 1.11
2447 reflections
191 parameters
22 restraints
H atoms treated by a mixture of independent and constrained refinement
Δρ_max_ = 0.38 e Å^−3^
Δρ_min_ = −0.45 e Å^−3^

### 

Data collection: *CAD-4 Software* (Enraf–Nonius, 1989 ▶); cell refinement: *CAD-4 Software*; data reduction: *XCAD4* (Harms & Wocadlo, 1995 ▶); program(s) used to solve structure: *SHELXS97* (Sheldrick, 2008 ▶); program(s) used to refine structure: *SHELXL97* (Sheldrick, 2008 ▶); molecular graphics: *SHELXTL* (Sheldrick, 2008 ▶); software used to prepare material for publication: *SHELXTL*.

## Supplementary Material

Crystal structure: contains datablocks global, I. DOI: 10.1107/S1600536809034540/hb5068sup1.cif

Structure factors: contains datablocks I. DOI: 10.1107/S1600536809034540/hb5068Isup2.hkl

Additional supplementary materials:  crystallographic information; 3D view; checkCIF report

## Figures and Tables

**Table 1:** Hydrogen-bond geometry (Å, °)

*D*—H⋯*A*	*D*—H	H⋯*A*	*D*⋯*A*	*D*—H⋯*A*
N1—H1*A*⋯O1^i^	0.845 (19)	1.99 (2)	2.782 (4)	156 (4)
N1—H1*C*⋯O2^ii^	0.89 (2)	1.87 (2)	2.752 (4)	169 (5)
N1—H1*B*⋯O1^iii^	0.872 (18)	1.885 (19)	2.749 (4)	171 (3)
O3—H3*B*⋯N1^iv^	0.82	2.54	3.267 (4)	149
C6—H6⋯O3^iii^	0.93	2.60	3.521 (4)	171

Symmetry codes: (i) 
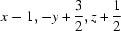
; (ii) 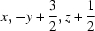; (iii) 
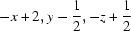
; (iv) 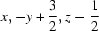.

**Fig. 1.**
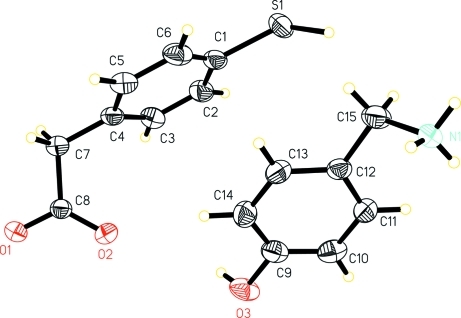
The molecular structure of (I) showing 35% probability displacement ellipsoids.

**Fig. 2.**
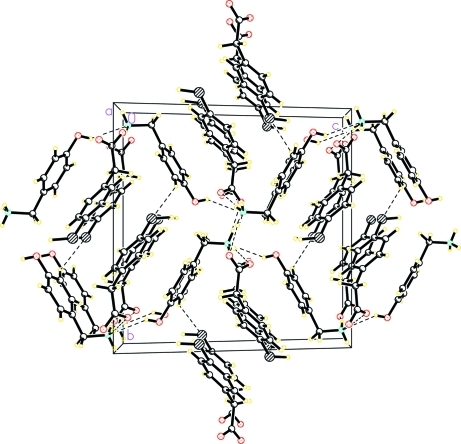
The packing of (I), showing intermolecular hydrogen bonds (dashed lines) along the a axis.

## supplementary crystallographic information

### Comment

There has been much research interest in the intermolecular interactions between carboxylic acid and amine. (Xia *et al.*, 2003; He *et al.*,  2008). The title compound (I) is presented in Fig.1, all bond lengths are within normal ranges (Allen *et al.*, 1987) (Table 1). The carboxylate cation and aminium anion are linked *via* N—H···O, O—H···N and C—H···O intermolecular hydrogen bonds (Table 2) into three network along the *a* axis. (Fig. 2).

### Experimental

A mixture of 2-(4-mercaptophenyl)acetic acid (336 mg, 2 mmol), and 4-(aminomethyl)phenol (246 mg, 2 mmol) was stirred in methanol (10 ml) for 1 h. After keeping the solution in air for 3 d, colourless blocks of (I) were formed.

### Refinement

All the H atoms, except for H1A, H1B and H1C attached to N1, H1D attached to S1, were placed in idealized positions (C—H = 0.93–0.97 Å, O—H = 0.82 Å) and refined as riding with *U*_iso_(H) = 1.2*U*_eq_(C) and *U*_iso_(H) = 1.5*U*_eq_(O). Atoms H1A, H1B and H1C and H1D were located from a difference map and their positions were freely refined.

### Crystal data

**Table:**

C_7_H_10_NO^+^·C_8_H_7_O_2_S^−^	*F*(000) = 616
*M**_r_* = 291.37	*D*_x_ = 1.391 Mg m^−^^3^
Monoclinic, *P*2_1_/*c*	Mo *K*α radiation, λ = 0.71073 Å
Hall symbol: -P 2ybc	Cell parameters from 1267 reflections
*a* = 6.545 (5) Å	θ = 2.4–24.4°
*b* = 14.792 (12) Å	µ = 0.24 mm^−^^1^
*c* = 14.868 (11) Å	*T* = 293 K
β = 104.78 (4)°	Block, colourless
*V* = 1391.8 (19) Å^3^	0.32 × 0.28 × 0.26 mm
*Z* = 4	

### Data collection

**Table:**

Enraf–Nonius CAD-4 diffractometer	2447 independent reflections
Radiation source: fine-focus sealed tube	1895 reflections with *I* > 2σ(*I*)
graphite	*R*_int_ = 0.061
ω/2θ scans	θ_max_ = 25.0°, θ_min_ = 2.0°
Absorption correction: ψ scan (North *et al.*, 1968)	*h* = −7→7
*T*_min_ = 0.927, *T*_max_ = 0.940	*k* = −17→17
6838 measured reflections	*l* = −14→17

### Refinement

**Table:**

Refinement on *F*^2^	Primary atom site location: structure-invariant direct methods
Least-squares matrix: full	Secondary atom site location: difference Fourier map
*R*[*F*^2^ > 2σ(*F*^2^)] = 0.054	Hydrogen site location: inferred from neighbouring sites
*wR*(*F*^2^) = 0.182	H atoms treated by a mixture of independent and constrained refinement
*S* = 1.11	*w* = 1/[σ^2^(*F*_o_^2^) + (0.1018*P*)^2^ + 0.3741*P*] where *P* = (*F*_o_^2^ + 2*F*_c_^2^)/3
2447 reflections	(Δ/σ)_max_ < 0.001
191 parameters	Δρ_max_ = 0.38 e Å^−^^3^
22 restraints	Δρ_min_ = −0.45 e Å^−^^3^

### Special details

**Table:**

Geometry. All e.s.d.'s (except the e.s.d. in the dihedral angle between two l.s. planes) are estimated using the full covariance matrix. The cell e.s.d.'s are taken into account individually in the estimation of e.s.d.'s in distances, angles and torsion angles; correlations between e.s.d.'s in cell parameters are only used when they are defined by crystal symmetry. An approximate (isotropic) treatment of cell e.s.d.'s is used for estimating e.s.d.'s involving l.s. planes.
Refinement. Refinement of *F*^2^ against ALL reflections. The weighted *R*-factor *wR* and goodness of fit *S* are based on *F*^2^, conventional *R*-factors *R* are based on *F*, with *F* set to zero for negative *F*^2^. The threshold expression of *F*^2^ > σ(*F*^2^) is used only for calculating *R*-factors(gt) *etc*. and is not relevant to the choice of reflections for refinement. *R*-factors based on *F*^2^ are statistically about twice as large as those based on *F*, and *R*- factors based on ALL data will be even larger.

### Fractional atomic coordinates and isotropic or equivalent isotropic displacement parameters (Å^2^)

**Table:**

	*x*	*y*	*z*	*U*_iso_*/*U*_eq_	
S1	0.76277 (18)	0.46123 (6)	0.14786 (7)	0.0712 (4)	
O1	1.3152 (3)	0.89080 (12)	−0.02444 (14)	0.0493 (5)	
O2	1.0949 (3)	0.85420 (13)	0.06100 (13)	0.0495 (5)	
C1	0.9143 (5)	0.53974 (18)	0.1055 (2)	0.0491 (7)	
C2	0.8159 (5)	0.59649 (19)	0.0354 (2)	0.0511 (7)	
H2	0.6711	0.5923	0.0096	0.061*	
C3	0.9338 (5)	0.65976 (19)	0.0038 (2)	0.0482 (7)	
H3A	0.8669	0.6986	−0.0438	0.058*	
C4	1.1530 (5)	0.66758 (18)	0.04140 (18)	0.0425 (6)	
C5	1.2476 (5)	0.60815 (19)	0.1105 (2)	0.0490 (7)	
H5	1.3931	0.6105	0.1354	0.059*	
C6	1.1284 (6)	0.54461 (19)	0.1435 (2)	0.0542 (8)	
H6	1.1935	0.5055	0.1912	0.065*	
C7	1.2790 (5)	0.73591 (19)	0.0050 (2)	0.0501 (7)	
H7A	1.2629	0.7240	−0.0606	0.060*	
H7B	1.4269	0.7273	0.0364	0.060*	
C8	1.2232 (4)	0.83435 (18)	0.01580 (18)	0.0385 (6)	
O3	0.6854 (4)	0.88722 (13)	0.17658 (14)	0.0603 (6)	
H3B	0.7023	0.8765	0.1248	0.090*	
N1	0.7229 (4)	0.56517 (17)	0.46681 (18)	0.0451 (6)	
C9	0.6981 (5)	0.8088 (2)	0.22552 (19)	0.051	
C10	0.5344 (5)	0.7868 (2)	0.2620 (2)	0.0560 (8)	
H10	0.4183	0.8249	0.2547	0.067*	
C11	0.5448 (5)	0.7067 (2)	0.3100 (2)	0.0494 (7)	
H11	0.4338	0.6897	0.3348	0.059*	
C12	0.7227 (4)	0.65084 (18)	0.32159 (17)	0.0423 (6)	
C13	0.8840 (5)	0.6771 (2)	0.28458 (19)	0.0479 (7)	
H13	1.0024	0.6402	0.2927	0.058*	
C14	0.8762 (5)	0.7568 (2)	0.23553 (19)	0.0525 (7)	
H14	0.9864	0.7744	0.2104	0.063*	
C15	0.7290 (6)	0.5601 (2)	0.3675 (2)	0.0561 (8)	
H15A	0.6097	0.5245	0.3334	0.067*	
H15B	0.8570	0.5290	0.3637	0.067*	
H1B	0.719 (5)	0.5117 (15)	0.4910 (19)	0.054 (9)*	
H1C	0.851 (5)	0.585 (4)	0.496 (3)	0.13 (2)*	
H1A	0.616 (4)	0.594 (2)	0.473 (3)	0.080 (13)*	
H1D	0.719 (10)	0.455 (4)	0.229 (2)	0.18 (2)*	

### Atomic displacement parameters (Å^2^)

**Table:**

	*U*^11^	*U*^22^	*U*^33^	*U*^12^	*U*^13^	*U*^23^
S1	0.1102 (8)	0.0416 (5)	0.0790 (6)	−0.0173 (5)	0.0555 (6)	−0.0050 (4)
O1	0.0561 (12)	0.0336 (11)	0.0632 (12)	−0.0046 (9)	0.0246 (10)	−0.0015 (9)
O2	0.0546 (12)	0.0400 (11)	0.0605 (12)	0.0055 (9)	0.0269 (10)	−0.0029 (9)
C1	0.074 (2)	0.0309 (15)	0.0500 (16)	−0.0040 (13)	0.0295 (15)	−0.0075 (12)
C2	0.0570 (18)	0.0426 (17)	0.0537 (16)	−0.0055 (13)	0.0144 (14)	−0.0068 (13)
C3	0.0542 (18)	0.0389 (16)	0.0494 (16)	0.0002 (13)	0.0092 (13)	0.0027 (12)
C4	0.0561 (17)	0.0292 (14)	0.0457 (14)	−0.0001 (12)	0.0192 (12)	−0.0055 (11)
C5	0.0552 (18)	0.0382 (15)	0.0513 (16)	0.0052 (13)	0.0097 (13)	−0.0038 (12)
C6	0.079 (2)	0.0376 (17)	0.0454 (15)	0.0083 (15)	0.0148 (15)	0.0061 (12)
C7	0.0582 (18)	0.0348 (15)	0.0645 (18)	0.0005 (13)	0.0290 (14)	−0.0042 (12)
C8	0.0390 (14)	0.0337 (14)	0.0422 (13)	−0.0005 (11)	0.0094 (11)	−0.0037 (11)
O3	0.0993 (17)	0.0344 (11)	0.0446 (11)	0.0015 (11)	0.0138 (11)	0.0139 (8)
N1	0.0511 (15)	0.0340 (13)	0.0545 (14)	−0.0007 (12)	0.0211 (12)	0.0041 (11)
C9	0.071	0.039	0.041	−0.002	0.011	0.000
C10	0.065 (2)	0.0479 (19)	0.0520 (16)	0.0133 (15)	0.0091 (14)	−0.0038 (14)
C11	0.0512 (17)	0.0486 (17)	0.0516 (16)	−0.0018 (14)	0.0192 (13)	−0.0057 (13)
C12	0.0546 (16)	0.0352 (14)	0.0374 (13)	−0.0009 (12)	0.0124 (12)	−0.0066 (11)
C13	0.0521 (17)	0.0464 (17)	0.0472 (15)	0.0062 (13)	0.0163 (13)	−0.0046 (12)
C14	0.0578 (18)	0.0570 (19)	0.0468 (15)	−0.0053 (15)	0.0208 (13)	−0.0036 (13)
C15	0.082 (2)	0.0366 (16)	0.0501 (16)	−0.0017 (15)	0.0187 (15)	−0.0030 (12)

### Geometric parameters (Å, °)

**Table:**

S1—C1	1.746 (3)	O3—H3B	0.8200
S1—H1D	1.31 (2)	N1—C15	1.490 (4)
O1—C8	1.266 (3)	N1—H1B	0.872 (18)
O2—C8	1.237 (3)	N1—H1C	0.89 (2)
C1—C2	1.365 (4)	N1—H1A	0.845 (19)
C1—C6	1.373 (5)	C9—C10	1.358 (5)
C2—C3	1.370 (4)	C9—C14	1.373 (5)
C2—H2	0.9300	C10—C11	1.376 (4)
C3—C4	1.405 (4)	C10—H10	0.9300
C3—H3A	0.9300	C11—C12	1.402 (4)
C4—C5	1.374 (4)	C11—H11	0.9300
C4—C7	1.491 (4)	C12—C13	1.366 (4)
C5—C6	1.389 (4)	C12—C15	1.501 (4)
C5—H5	0.9300	C13—C14	1.380 (4)
C6—H6	0.9300	C13—H13	0.9300
C7—C8	1.520 (4)	C14—H14	0.9300
C7—H7A	0.9700	C15—H15A	0.9700
C7—H7B	0.9700	C15—H15B	0.9700
O3—C9	1.361 (4)		
			
C1—S1—H1D	131 (3)	C15—N1—H1C	104 (4)
C2—C1—C6	121.0 (3)	H1B—N1—H1C	102 (4)
C2—C1—S1	118.9 (3)	C15—N1—H1A	112 (3)
C6—C1—S1	120.0 (2)	H1B—N1—H1A	107 (3)
C1—C2—C3	119.0 (3)	H1C—N1—H1A	119 (5)
C1—C2—H2	120.5	C10—C9—O3	118.1 (3)
C3—C2—H2	120.5	C10—C9—C14	123.9 (3)
C2—C3—C4	121.8 (3)	O3—C9—C14	118.0 (3)
C2—C3—H3A	119.1	C9—C10—C11	118.3 (3)
C4—C3—H3A	119.1	C9—C10—H10	120.9
C5—C4—C3	117.7 (3)	C11—C10—H10	120.9
C5—C4—C7	121.3 (3)	C10—C11—C12	120.1 (3)
C3—C4—C7	121.0 (3)	C10—C11—H11	120.0
C4—C5—C6	120.7 (3)	C12—C11—H11	120.0
C4—C5—H5	119.6	C13—C12—C11	119.1 (3)
C6—C5—H5	119.6	C13—C12—C15	120.2 (3)
C1—C6—C5	119.8 (3)	C11—C12—C15	120.6 (3)
C1—C6—H6	120.1	C12—C13—C14	121.9 (3)
C5—C6—H6	120.1	C12—C13—H13	119.1
C4—C7—C8	116.2 (2)	C14—C13—H13	119.1
C4—C7—H7A	108.2	C9—C14—C13	116.8 (3)
C8—C7—H7A	108.2	C9—C14—H14	121.6
C4—C7—H7B	108.2	C13—C14—H14	121.6
C8—C7—H7B	108.2	N1—C15—C12	113.7 (2)
H7A—C7—H7B	107.4	N1—C15—H15A	108.8
O2—C8—O1	124.8 (2)	C12—C15—H15A	108.8
O2—C8—C7	120.0 (2)	N1—C15—H15B	108.8
O1—C8—C7	115.2 (2)	C12—C15—H15B	108.8
C9—O3—H3B	109.4	H15A—C15—H15B	107.7
C15—N1—H1B	112 (2)		
			
C6—C1—C2—C3	−0.8 (4)	C4—C7—C8—O1	−172.5 (2)
S1—C1—C2—C3	178.3 (2)	O3—C9—C10—C11	178.8 (2)
C1—C2—C3—C4	0.2 (4)	C14—C9—C10—C11	−1.6 (5)
C2—C3—C4—C5	1.2 (4)	C9—C10—C11—C12	1.0 (4)
C2—C3—C4—C7	179.0 (3)	C10—C11—C12—C13	0.0 (4)
C3—C4—C5—C6	−1.9 (4)	C10—C11—C12—C15	−175.3 (3)
C7—C4—C5—C6	−179.8 (3)	C11—C12—C13—C14	−0.5 (4)
C2—C1—C6—C5	0.0 (4)	C15—C12—C13—C14	174.8 (3)
S1—C1—C6—C5	−179.0 (2)	C10—C9—C14—C13	1.1 (4)
C4—C5—C6—C1	1.3 (4)	O3—C9—C14—C13	−179.3 (2)
C5—C4—C7—C8	−119.7 (3)	C12—C13—C14—C9	0.0 (4)
C3—C4—C7—C8	62.5 (4)	C13—C12—C15—N1	120.6 (3)
C4—C7—C8—O2	7.1 (4)	C11—C12—C15—N1	−64.2 (4)

### Hydrogen-bond geometry (Å, °)

**Table:**

*D*—H···*A*	*D*—H	H···*A*	*D*···*A*	*D*—H···*A*
N1—H1A···O1^i^	0.85 (2)	1.99 (2)	2.782 (4)	156 (4)
N1—H1C···O2^ii^	0.89 (2)	1.87 (2)	2.752 (4)	169 (5)
N1—H1B···O1^iii^	0.87 (2)	1.89 (2)	2.749 (4)	171 (3)
O3—H3B···N1^iv^	0.82	2.54	3.267 (4)	149
C6—H6···O3^iii^	0.93	2.60	3.521 (4)	171

Symmetry codes: (i) *x*−1, −*y*+3/2, *z*+1/2; (ii) *x*, −*y*+3/2, *z*+1/2; (iii) −*x*+2, *y*−1/2, −*z*+1/2; (iv) *x*, −*y*+3/2, *z*−1/2.
